# Post-Traumatic Stress Disorder and Alcohol Consumption: Biological Mechanisms of Stress Resilience to Subsequent Alcohol Consumption

**DOI:** 10.35946/arcr.v46.1.01

**Published:** 2026-01-13

**Authors:** Quilla C. Flanagan-Burt, Celia Middleton, Junghyup Suh

**Affiliations:** Basic Neuroscience Division, McLean Hospital, Belmont, Massachusetts. Department of Psychiatry, Harvard Medical School, Boston, Massachusetts

**Keywords:** alcohol, alcohol use disorder, animal models, neural circuitry, post-traumatic stress disorder, resilience, stress, susceptibility

## Abstract

**PURPOSE:**

Resilience is crucial in mitigating the risk of stress-related health issues. Although many people can adapt to adverse stress or trauma, stress exposure can increase the risk of health issues, including obesity, cardiovascular disease, and digestive illnesses. Some individuals may even develop debilitating conditions, such as post-traumatic stress disorder (PTSD). People with PTSD often struggle to adapt, sometimes turning to alcohol to cope, which can lead to alcohol use disorder (AUD), characterized by excessive alcohol-seeking and dependence. Understanding the biological underpinnings of resilience, therefore, is a key to preventing both PTSD and AUD. Recent research has uncovered the neurobiological traits that protect against the development of stress-induced alcohol dependence. Studies have shown that proactive coping and a lack of stress-related symptoms are associated with resilience. Preclinical studies, especially in rodents, have provided deeper insights into how stress impacts alcohol-seeking behaviors. Research has shown that resilience involves adaptive changes at the molecular, cellular, neural circuit, and systems levels. This review aims to integrate this research to better understand what makes people vulnerable to stress and alcohol consumption, highlighting aspects frequently overlooked in clinical models.

**SEARCH METHODS:**

This review employed systematic search strategies to achieve a comprehensive and structured assessment of the existing body of literature using the academic databases PubMed, Google Scholar, and Web of Science. Targeted keywords included “stress,” “PTSD,” “trauma,” “alcohol,” “AUD,” “resilience,” “vulnerability,” “susceptibility,” “sex difference,” and “animal models”; Boolean operators (AND, OR, NOT) were used to refine the results. Exclusion criteria included research published before 1990, research that was not peer reviewed, and publications in languages other than English. Additional studies were identified by reviewing the references cited in key articles as well as by identifying subsequent studies that referenced these pivotal works. The search was carried out from April to June 2025 focusing on, but not limited to, experiments involving rodent models.

**SEARCH RESULTS:**

The search yielded a total of 347 articles. After screening, 283 articles were removed, either because they met the predefined exclusion criteria or because they were duplicates. This process resulted in 64 articles, forming the core of this comprehensive review.

**DISCUSSION AND CONCLUSIONS:**

This review summarizes an overview of recent progress in studies of PTSD and AUD, primarily focusing on the effect of resilience on post-stress alcohol intake in animal models. The findings highlight several biomarkers that may help identify individuals at risk for PTSD, AUD, or their co-occurrence, acknowledging that no single identifier can predict post-trauma outcomes. The identification of these markers is an ongoing process, yet it will be crucial for early diagnosis and risk assessment moving forward.

**KEY TAKEAWAYS**
- Susceptible versus resilient phenotypes appear across multiple preclinical models of stress, including stress periods in both early life and adulthood.
- There are distinct sex differences in reactivity to stress and subsequent alcohol consumption behaviors.
- Enhanced connectivity and neuroanatomic integrity in essential brain circuits may underlie resilience to stress and provide potential targets for therapeutic intervention.
- Variations in neuroendocrine responses and receptor expression in the hypothalamic-pituitary-adrenal axis may serve as biomarkers for identifying individuals at risk for developing alcohol use disorder after experiencing stress and post-traumatic stress disorder.

Post-traumatic stress disorder (PTSD) is a debilitating psychiatric disorder that can develop after a person experiences or witnesses a traumatic event. It is often characterized by hyperarousal, anxiety, negative emotions and thoughts, avoidance, sleep disturbance, and re-experiencing of the traumatic event.^1^–^3^ Although approximately 70% of people in the U.S. population will encounter at least one traumatic event in their lifetime, only roughly 7% will develop PTSD at some point, with about 4% experiencing it in the past year; women (5%) are twice likely as men (2%) to develop the condition in a given year.^4^, ^5^ Research has shown that the risk of developing PTSD is multifactorial and is influenced by genes, the environment, and individual history.^2^,^6^–^9^ However, traditional assessments often have concentrated on trauma exposure and the development of PTSD, potentially neglecting a broader spectrum of stress-related issues. These may encompass various mental health conditions that may exist independently of PTSD, including depression, anxiety disorders, and alcohol use disorder (AUD), as well as a variety of physical health issues, such as obesity, hypertension, cardiovascular disease, and digestive disorders.^2^, ^10^ Therefore, understanding the mechanisms of resilience—the biological factors that enable some individuals to navigate adversity without succumbing to these diverse stress-related sequelae—becomes paramount. This review seeks to elucidate some of the differences that may underlie susceptible versus resilient phenotypes in response to trauma. In this context, resilience is defined as the ability to thrive or the process of effectively adapting in response to significant adversity, trauma, or other substantial causes of stress that lead to PTSD-like symptoms.^11^–^14^

For individuals suffering from PTSD-related symptoms, the development of comorbid substance misuse is not uncommon. People with PTSD who go on to develop severe negative symptomology often engage in binge drinking or heavy drinking in a repeated manner. The National Institute on Alcohol Abuse and Alcoholism defines binge drinking as consuming enough alcohol to bring a person’s blood alcohol concentration to 0.08% or above, typically four or more drinks for women and five or more drinks for men in about 2 hours. Heavy drinking is defined as consuming more than five drinks on any day or 15 drinks per week for men, or more than four drinks on any day or eight drinks per week for women.^15^ The aforementioned alcohol drinking patterns can lead to comorbid AUD.^1^ AUD can be also characterized by excessive alcohol seeking as well as physiological and psychological dependence on alcohol.^1^ Clinical and epidemiological studies have repeatedly indicated that PTSD is linked to a threefold increased chance of developing AUD, with an estimated lifetime prevalence of AUD reaching 40% among individuals with PTSD.^10^,^16^,^17^ Therefore, resilience appears to be crucial in mitigating the risk for PTSD and subsequent AUD. Recent clinical research has begun to identify the neurobiological traits that provide protection against alcohol dependence following stress exposure.^2^ Proactive coping mechanisms to face the stressor and lack of stress-related symptoms have been consistently linked to resilience against the stress-induced enhancement of alcohol effects and a decreased risk of AUD.

However, the effect of stress on the initiation, maintenance, and relapse to alcohol misuse remains understudied. Therefore, preclinical studies using rodent models have begun to explore the effects of stress on subsequent alcohol seeking and consumption by examining the behavioral and physiological characteristics that define animals’ resilience to stressors.^1^, ^18^ In clinical contexts, resilient individuals often display a range of psychological and social characteristics, including positive emotions, realistic optimism (i.e., balancing a hopeful perspective with a grounded understanding of reality), proactive stress coping strategies, and healthy lifestyles.^19^ Interestingly, resilience is a dynamic process, and an individual may exhibit resilience to certain adverse events while being more susceptible to others. Furthermore, many resilience factors may be learnable, and individuals can undergo training to enhance their resilience.^3^, ^11^ The findings from rodent studies are unraveling the neurobiological substrates of resilience and are essential for developing pharmacological and psychological strategies to improve stress resilience and avert the onset of alcohol dependence and misuse. This review will first discuss the protective variables linked to resilience, such as neuroendocrine responses to stress. Subsequently, it will examine various animal models employed to investigate resistance to stress and trauma and evaluate progress on the neurobiological substrates of resilience, including molecular and cellular adaptations, neuroimmune alterations and brain circuit changes. Finally, it will explore the sex differences in stress responses, coping strategies, and subsequent alcohol drinking behaviors.

## Search Methods

This literature review employed systematic search strategies to achieve a comprehensive and structured assessment of the existing body of literature. Literature searches were conducted using the academic databases PubMed, Google Scholar, and Web of Science to mitigate potential bias and maximize the chances of capturing relevant studies across different research domains. Targeted keywords, such as “stress,” “PTSD,” “trauma,” “alcohol,” “AUD,” “resilience,” “vulnerability,” “susceptibility,” “sex difference,” and “animal models” were used alongside Boolean operators (AND, OR, NOT) to refine the results. Exclusion criteria included research published before 1990, research that was not peer reviewed, experiments conducted in nonrodent animal models, and publications in languages other than English to ensure reliability and relevance and to identify key publications. Further studies were identified by reviewing the references cited in key articles as well as by identifying subsequent studies that referenced these pivotal works. This approach ensured the inclusion of both foundational and recent research. The literature search was completed between April and June 2025, yielding a total of 347 articles. After applying the exclusion criteria and removing 283 articles that either did not fit the predetermined criteria or were duplicates, 64 articles remained, forming the core of this comprehensive review. Additionally, human literature pertinent to the focus of the current review has been also included under these criteria.

## Results

### Stress Resilience

Many individuals exposed to stress demonstrate remarkable adaptability and manage to overcome adversity while preserving normal psychological and physical functions. Nevertheless, the observation that most people do not develop stress-related illnesses necessitates a thoughtful and nuanced reevaluation. Traditional clinical investigations on PTSD using recognized rating scales have concentrated on determining the biological foundations of stress resilience, revealing the protective roles of genetic, developmental, cognitive, psychological, and neurobiological factors.^11^–^14^ For example, neuroimaging studies showed that the activity in several brain regions reflected the protective effect of resilience against adverse stress outcomes.^8^ Additionally, preclinical studies have identified various neurotransmitter, neuropeptide, hormone, immune system, and epigenetic changes as correlating with stress resilience.^11^,^12^,^20^

These findings, however, need to be reconciled with the broader notion that stress-related issues extend beyond PTSD to include other mental health conditions, such as depression, anxiety disorders, and AUD, that may not be associated with PTSD symptoms. Therefore, rather than merely avoiding the harmful consequences of stress, more recent studies have suggested that resilience to stress is an active process. Active coping methods^20^—including the development of social support and friendships, exercising self-control, developing a strong sense of identity, and developing a sense of purpose—are associated with lower reactivity in the hypothalamic-pituitary-adrenal (HPA) axis (i.e., the primary system controlling the body’s neuroendocrine stress response) and higher reactivity in the sympathetic nervous system to stress exposure.^21^ In contrast, passive coping is marked by dependence on others to mitigate stress and feelings of helplessness, as well as high HPA axis reactivity.^20^, ^22^ Therefore, resilience is primarily facilitated by unique adaptations at the social, behavioral, neuronal circuit, and molecular levels that mitigate passive or maladaptive responses to stress. The ongoing search for biomarkers for resilience offers a promising avenue for developing targeted treatments not only for stress-related disorders, such as PTSD, but also for the commonly co-occurring AUD. Such treatments would ultimately support individuals vulnerable to the full spectrum of negative health outcomes related to aversive stress and trauma.

### Rodent Models of PTSD and Alcohol Drinking

The increasing recognition of stress resilience in humans has prompted a corresponding examination in preclinical models. Rodent models have proven crucial in facilitating analyses of brain structures and neural circuits, as well as for investigating behavioral characteristics associated with stress-related disorders, such as PTSD. These animal models enable researchers to simulate symptoms of PTSD in controlled settings, offering insights into the underlying mechanisms and potential interventions. Although many stressors can elicit behavioral and physiological responses akin to PTSD symptoms, prevalent rodent models often utilize acute stressors to independently study stress susceptibility and resilience and to reduce the likelihood of subjects developing depression-related behaviors (see Table 1 for details). Because PTSD is developed following stressful or traumatic experiences, it is worth noting that some of the methodologies listed below are also used in general studies of stress and anxiety beyond the scope of PTSD. Additionally, researchers will often combine two or more methodologies to create the desired PTSD-specific behavioral phenotypes.

#### Predator stress

Predator stress models are widely used; for this approach, animals are placed in a new context and exposed to a predator-related odor, such as bobcat urine, for either a single or repeated exposure. These paradigms leverage the rodent’s natural instincts to reliably induce high arousal states, while increasing startle reactivity and anxiety-like behaviors.^23^, ^24^ The response to predator-related stressor varies among individuals and across sexes. In a study in which male and female adult Wistar rats were exposed to bobcat urine and tested for contextual avoidance 24 hours later, a subset of animals exhibited an increase in ethanol intake relative to their prestress baseline. This subset primarily included those animals whose behavior was also characterized by heightened anxiety or specific stress-coping behavioral strategies, such as active avoidance of the odor.^24^ In alcohol-drinking male rats, predator odor exposure increased startle responses in an acoustic startle task, whereas stressed females showed blunted startle responses relative to female controls and stressed males, regardless of prior ethanol consumption.^24^ Therefore, the model offers opportunities to investigate biological underpinnings of sex-specific, stress-related behavioral heterogeneity and the impact of stress and alcohol exposure on subsequent alcohol drinking. However, the model generally emphasizes anxiety measures, often overlooking cognitive symptoms, such as memory impairment, a component that is central to the PTSD symptom profile.

#### Stress-enhanced fear learning

A more recent PTSD model is stress-enhanced fear learning (SEFL). In this paradigm, animals are first exposed to an intense sensitizing stressor, such as foot shocks, that does not cause fear on its own. A later, single mild fear-conditioning trial then produces an exaggerated fear response. This maladaptive expression of fear in a safe environment and inability to discriminate between a threatening context or safe context mirrors the heightened overgeneralization of fear responding and extinction-resistant traumatic memory that are essential cognitive symptoms of PTSD.^25^, ^27^ Previous studies demonstrated that inbred mice (C57BL/6) subjected to an adapted SEFL paradigm could be categorized by an unbiased cluster analysis into two low- and high-freezing groups during fear extinction to tone. This particular SEFL model induced stress via 2-hour restraint, which was followed 7 days later by standard tone-shock fear conditioning, 4 days later by extinction training, and fear-memory retrieval testing 30 days after the tone shock.^25^ Male mice with prior stress exposure showed extinction resistance compared to the control male group without stress exposure. However, across both groups, animals with lower freezing rates during fear conditioning exhibited typical extinction, whereas those with high freezing rates were resistant to extinction and more susceptible to stress, correlating with PTSD-like symptoms. In contrast, female mice previously exposed to restraint stress displayed more freezing during fear conditioning than their control counterparts without stress exposure, but both groups showed comparable extinction resistance. These observations suggest that incubated stress may influence fear conditioning differently across sexes.^25^ RNA sequencing of the basolateral amygdala revealed transcriptional divergence between the susceptible and resilient groups of male mice.^25^ Particularly, the study identified differences in genes linked to neurotransmitters and their receptors and to genetic variations associated with PTSD, including pituitary adenylate cyclase-activating polypeptide (*Adcyap1*), brain-derived neurotrophic factor (*Bdnf*), and dopamine D2 receptor (*Drd2*).^35^–^37^ Genetic association studies have also implicated a role for these genes in alcohol craving, consumption, and dependence.^38^–^40^

A separate study demonstrated that a less-severe SEFL procedure resulted in distinct groups of stress susceptible and resilient rats that exhibited differences across various measures of PTSD-like symptomatology.^26^ Both male and female susceptible rats showed heightened fear generalization and impaired fear extinction. Furthermore, susceptible male rats without prior alcohol exposure increased alcohol intake and preference after a stress experience; this was not observed in susceptible female rats. However, susceptibility did not seem to influence alcohol-related behaviors after stress in both male and female rats that had previous alcohol exposure.^26^ These results suggest that alcohol access following a stress experience may blunt potential differences in subsequent alcohol intake between susceptible and resilient subjects in a sex-dependent manner.

#### Inhibitory avoidance

PTSD involves more than just Pavlovian conditioning (i.e., a response to only a specific stimulus or stressor); it also includes fear overgeneralization, nonassociative hyperarousal, and fear. Therefore, procedures incorporating non-Pavlovian attributes, such as inhibitory avoidance, offer advantages over pure classical conditioning. “Two-hit” inhibitory avoidance effectively generates PTSD-like symptoms by integrating both operant and Pavlovian learning under conflict.^41^ This foot-shock–based paradigm involves an initial shock when the animal enters a dark chamber from an illuminated one, followed by a second shock either in the same (familiar [FAM]) or a novel (NOV) context, effectively generating PTSD-like symptoms. Previous studies reported that the impacts of NOV versus FAM stress on subsequent voluntary ethanol consumption were sex-dependent in adult Wistar rats.^27^ For example, FAM stress increased ethanol intake in a two-bottle choice paradigm in males, whereas NOV stress increased ethanol intake in females. Both stressors combined increased ethanol preference across sexes.^27^ Sex differences also appeared across a number of anxiety-related measures. Male rats in both the NOV and FAM condition showed an increase in the time spent in the open arms of an elevated plus maze, a behavioral measure often used to evaluate locomotor activity and anxiety levels. This may suggest a generalization of fear across contexts—that is, the animals transfer the fear from the dark inhibitory avoidance chamber to the dark closed arms of the maze so that the open arms of the maze appear to serve as the less aversive option, leading to a false “lower anxiety” phenotype.^27^ However, when rats had intermittent ethanol access (Monday, Wednesday, and Friday; 24 hours/day) for 3 weeks before the initial shock of the “two-hit” model, a second shock in the NOV context reduced ethanol intake in male rats, which had increased after the inhibitory avoidance procedure in ethanol-naïve rats. Similar to the previous experiment involving ethanol-naïve rats, FAM males with prior ethanol exposure spent more time in the open arms and the center of the maze compared to control males, indicating a form of fear overgeneralization. However, there were no significant differences in female performance across conditions. These results emphasize the importance of trauma context and sex differences, and point to a role of prior ethanol exposure in modulating vulnerability to stress-induced ethanol escalation.

#### Social defeat stress

As social stressors and anxiety have been extensively implicated as a significant risk factor for increased substance and alcohol use in humans, social defeat stress (SDS) models are similarly employed to examine the impacts of chronic social stress and its effects on subsequent alcohol consumption in rodents.^29^,^42^,^43^ SDS models leverage natural behaviors in mice, using aggressive CD-1 mice to repeatedly defeat the less aggressive C57BL/6 mice (which are the subject of the study) across multiple social confrontations.^30^ This stress is continued following the brief confrontation bout by “cohousing” the animals in the same cage but separating them using a perforated plastic divider. As a result, the subject animals continue to be exposed to the aggressor animal and its odor without physical contact.^30^ Protocols have been established for both 10- and 21-day defeat cycles, in which the subject is exposed to a different aggressor animal each day.^30^, ^31^ Animals exposed to SDS not only exhibit distinct behavioral changes, including heightened anxiety and depression-like symptoms, but also demonstrate escalated alcohol consumption.^29^ Like other stress models, SDS models also have revealed variations in stress responses, distinguishing between susceptible and resilient subgroups. Resilient animals showed protective responses, including attenuated stress responses under social stress conditions and reduced alcohol intake, whereas susceptible animals exhibited heightened vulnerability to social stress and an increase in alcohol consumption.^29^

#### Early life stress—Limited bedding and nesting

In many clinical cases, individuals who are diagnosed with PTSD and substance use disorders had experienced stressors or traumas in early life. Research has indicated that exposure to trauma during developmental periods differentially impacts health outcomes when compared to exposure during adulthood.^44^, ^45^ Thus, it is crucial to develop additional preclinical models to mimic traumatic experiences during development and study the long-term impacts on physiological responses to later stress and on brain reward systems. The limited bedding and nesting (LBN) models are a variation of early life stress that leverage bedding resource scarcity to evoke erratic maternal behavior.^32^ LBN models have been used in both rats and mice. Typically, the cage is fitted with a wire mesh insert that suspends the dam and pups above the bedding; only a portion of nestlet is provided above the mesh.^46^ The resulting maternal stress is reflected in decreased quality of maternal care and changes in patterns of care behavior, which in turn creates a stressful environment for the litter. In rats, the inability of the dam to construct a desirable nest resulted in elevated basal corticosterone levels in pups by postnatal day 9 that persisted into adulthood.^46^ Hypertrophied adrenal glands were also observed in pups of outbred rat strains, which have greater genetic variability than inbred rats, including Sprague Dawley and Long-Evans.^32^ Pups with LBN exposure demonstrated cognitive deficits, impaired social functions, increased anxiety, anhedonia, altered gut function, and, most critically, vulnerability to future stress.^32^ For example, mice exposed to LBN stress displayed suppressed emotional responses in contexts previously paired with an aversive stimulus, such as a shock, a full week earlier in development than their control counterparts without LBN stress (postnatal day 22 versus postnatal days 28–35).^32^, ^47^ Alterations of fear circuits appear to persist into adulthood, because LBN mice showed a decreased ability to distinguish between the presentation of a conditioned stimulus (cue-on) and intervals between the cues (safe periods) following fear learning.^32^, ^48^

Several studies evaluated the effect of LBN stress on subsequent alcohol intake. One study reported that mice with LBN experience showed accelerated alcohol intake in male mice and exacerbated affective disturbance during withdrawal; however, females were insensitive to these effects.^49^ In contrast, a separate study showed that the LBN alone increased alcohol intake only in female adolescent rats.^50^

#### Early life stress—Maternal deprivation

Another frequently used model of early life stress is the maternal deprivation model. Deprivation can either be a single bout or repeated daily separation between the dam and pups.^32^ In a study using male and female Wistar rats, pups experienced a single 24-hour separation from the dam on postnatal day 9, before undergoing a voluntary alcohol intake procedure (two-bottle choice) during adolescence. Maternal deprivation alone did not change baseline voluntary alcohol consumption in adolescence. However, a period of alcohol withdrawal and a secondary stressor (restraint) induced an escalation in their alcohol consumption.^33^ This indicates that early life stress such as maternal deprivation may increase susceptibility to increased alcohol consumption in later life following secondary stress exposure.^51^

The use of a repeated separation model (180 minutes of maternal deprivation from postnatal days 1–14) in C57BL/6J mice induced changes in adult alcohol consumption. For example, in comparison to their control counterparts, female mice that had experienced maternal deprivation exhibited increased alcohol seeking during week 3 of an operant self-administration procedure, where responses were reinforced under a fixed ratio 5 schedule (i.e., five nose pokes were required to deliver ethanol solution).^51^ Additionally, both male and female mice that had experienced maternal deprivation showed higher breakpoints in 2-hour progressive ratio tests, wherein animals cease responding to an increasing requirement of nose pokes following each reward.^51^ Although maternal deprivation stress produced varying results due to paradigm specifics and species, it is a valuable tool for studying early life stress and its impacts on future behavior, including alcohol consumption.^33^

#### Chronic restraint stress

Research utilizing a two-hit model of early life stress, starting with maternal deprivation in infancy followed by chronic restraint stress (CRS) in adolescence, has provided insights into how adolescent environmental factors shape adult stress responses.^34^ In this model, adult male Sprague Dawley rats previously exposed to maternal deprivation exhibited resilience, with no significant changes in corticosterone levels at baseline or during CRS, whereas nonmaternal deprivation males demonstrated elevated corticosterone levels in response to CRS.^34^ In contrast, adult females exposed to maternal deprivation, despite having lower baseline corticosterone levels than controls, exhibited a significant increase in corticosterone levels following CRS, returning to levels comparable to nonmaternal deprivation controls. This response suggests a compensatory mechanism to mitigate the effects of early life stress, demonstrating a form of resilience emerging from chronic hyperactivation of the HPA axis, a mechanism often implicated in stress-related conditions, such as PTSD.

Complementary findings came from a recent study examining whether environmental enrichment characterized by increased stimuli and social interaction during adolescence could enhance resilience to social stress.^52^ Susceptible mice showed altered levels of cytokine interleukin-6 and chemokine C-X3-C motif ligand 1 (CX3CL1). When an enriched environment was provided, susceptible mice exhibited decreases in ethanol consumption and levels of inflammatory markers.^52^ These findings suggest that adolescent environmental enrichment may provide a protective effect against the behavioral and neuroinflammatory responses following social stress.^52^ Together, these studies suggest that early life environmental factors may influence responses to later-life stress and point to potential areas for therapeutic intervention for individuals at risk of developing PTSD.

### Susceptible Versus Resilient Ethanol Consumption

Following exposure to a stress paradigm, animals can be categorized into susceptible or resilient phenotypes regarding their ethanol consumption (see Table 2). These phenotypes fall along a spectrum, with stress response acting as a critical determinant of subsequent drinking behavior. The use of stress response as a predictor of future drinking patterns has the potential to inform future identification of individuals with PTSD who are most at risk for development of AUD. While thresholds for defining susceptible versus resilient phenotypes vary among researchers, there are a few common conventions. Animals can be categorized as susceptible if they exhibit an increase in ethanol consumption of 20% or more after a predator stressor exposure.^23^ In a study using a repeated intermittent exposure following the first predator stressor, 24% of male animals and 20% of female animals showed an increase of 20% or more relative to baseline ethanol consumption.^23^ In susceptible mice, ethanol intake escalated progressively after multiple stress exposures.^23^ The resilient subgroup exhibited a contrasting trend with a consistent decrease in ethanol intake following repeated stress exposure, suggesting an ability to resist stress-induced increases in ethanol consumption. In the susceptible subgroup, baseline ethanol intake was consistently lower in both males and females compared to the resilient group. Moreover, mice with lower baseline drinking prior to stress were more likely to significantly increase ethanol intake after stress.^23^ Resilient animals exhibited stable and higher baseline drinking levels, showing minimal to no increase in ethanol intake after stress, suggesting inherent differences in ethanol use prior to stress exposure.^23^ These observations suggest that baseline drinking may be predictive of post-stress drinking behavior.

Significant sex-dependent variations exist in the response to stress that may influence alcohol consumption. For example, females typically exhibit higher baseline and stress-induced corticosterone levels than males. In a predator stress model, females demonstrated significantly elevated corticosterone levels relative to males immediately after stress exposure, as well as at 30 and 60 minutes post-stress.^24^ The heightened hormonal response may amplify female stress susceptibility, potentially driving behavioral changes, such as increased ethanol consumption.^23^ Behavioral studies have further illustrated these dynamics using the elevated plus maze.^24^ In a study using rats exposed to predator stress, female rats with a history of alcohol drinking exhibited more time spent in the open arms of the maze and increased entries into the open and closed arms compared to males. This pattern of behaviors indicates not only reduced anxiety but also greater overall locomotor activity, suggesting that stress and alcohol history may interact to change anxiety-like behaviors in a sex-dependent manner.^24^ Male and female rats could also be further subcategorized into avoiders and nonavoiders based on the proportion of time spent in the chamber paired with previous exposure to predator odor.^24^ Although in this study sex differences in alcohol consumption were not significant across drinking sessions, the proportion of male avoiders that met stress reactivity criteria was 27% higher than females.^24^ This suggests that females may respond to stress differently in terms of emotional processing and hormonal responses, which could potentially influence patterns of ethanol consumption. Together, these findings emphasize the importance of considering sex differences in stress reactivity and their implications for alcohol use behaviors.

### Biological Substrates of Resilience

#### Brain circuits

Individual differences in stress susceptibility and resilience have been linked to distinct neuroanatomic patterns across key brain regions involved in stress regulation. For example, apart from its roles in learning and memory, the hippocampus has been well studied as a brain structure strongly tied to stress-related disorders, such as depression and PTSD. Previous studies observed lower expression of two immediate early genes— activity-regulated cytoskeleton-associated protein *(Arc)* and early growth response 1 *(Egr1)*—in the ventral hippocampus (vHPC) of mice that showed resilience to chronic SDS.^58^, ^59^ Because *Arc* and *Egr1* are involved in synaptic plasticity, reduced expression of these genes could indicate less engagement of stress-related circuits in the hippocampus of resilient animals. Furthermore, resilient mice displayed an increase in paired pulse response, which reflects presynaptic alterations in the probability of neurotransmitter release, in vHPC projections to the nucleus accumbens (NAc), indicating reduced glutamate release from hippocampal afferents.^58^ In contrast, high levels of glutamate transmission in the NAc have been implicated in animal models of depression following chronic SDS.^58^ In addition, the induction of long-term depression in this pathway promoted resilience.^58^ NAc glutamate transmission may therefore be mediating alterations in synaptic plasticity and subsequently lead to changes in reward and motivation behaviors following acute or chronic stress experiences.

In contrast, enhanced hippocampal activity has been observed in stress-susceptible animals. Chronic mild stress-induced reduction of the number of inhibitory interneurons in the vHPC that express somatostatin, neuropeptide Y (NPY), or calretinin appeared to drive an increase in the hippocampal activity in rats.^60^ A recent study also demonstrated that the activation and inhibition of parvalbumin-expressing interneurons in the dentate gyrus promoted susceptibility and resilience to chronic SDS, respectively.^61^ The vHPC and its connectivity to the NAc are also involved in alcohol drinking.^61^ A recent study showed that inhibition of the vHPC via a chemogenetic approach resulted in no changes in alcohol drinking in alcohol-dependent C57BL/6J mice but increased alcohol drinking in nondependent mice. In contrast, chemogenetic activation of vHPC-NAc projections decreased alcohol drinking in the alcohol-dependent mice with no effects on the nondependent mice.^62^ These findings suggest that higher activity in the vHPC via reduced inhibitory tone in local circuits and enhanced functional connection between the vHPC and NAc are linked to stress susceptibility and an escalation in alcohol consumption following stress.

Growing evidence also has indicated a crucial function of brain reward systems in stress resilience and susceptibility. Previous studies demonstrated that the ventral tegmental area (VTA), a brain region primarily associated with dopamine and reward, is involved in resilience and susceptibility to SDS.^63^ Moreover, phasic stimulation of VTA neurons targeting the NAc, rather than the medial prefrontal cortex (mPFC), resulted in susceptibility to SDS in mice. Conversely, optogenetic inhibition of the VTA-NAc projection enhanced resilience, whereas the suppression of the VTA-mPFC projection fostered susceptibility.^64^ At a macroscopic level, a study using volumetric magnetic resonance imaging reported that susceptibility to chronic SDS was associated with increased volumes of the VTA, amygdala, hippocampus, and hypothalamus, and with smaller volumes of the cingulate cortex and NAc in mice.^53^ These structural changes align with the respective roles of these brain regions in reward processing, emotional reactivity, and neuroendocrine regulation that have been observed in patients with stress-related disorders.^2^ Other studies in mice found that the VTA and amygdala volumes correlated positively with social avoidance, suggesting heightened activity in stress-promoting circuits, whereas smaller cingulate cortex and NAc volumes reflect reduced regulatory and reward-modulating ability.^62^ Structural covariance analyses further revealed that in susceptible mice, the VTA and cingulate cortex exhibited opposing changes in volume, reflecting negative interactions between these regions. Conversely, resilience appeared to be associated with balanced structural patterns and stronger connectivity between regions like the hippocampus and hypothalamus.^53^ In addition to the volumetric analyses, the study also used fractional anisotropy, a measure of the directionality of water diffusion in the brain, measured from diffusion-weighted imaging. These analyses showed that fractional anisotropy correlated inversely with volume in susceptible regions, indicating that stress-induced structural changes may lead to the impaired functional connectivity and subsequently susceptible phenotypes.^53^ Together, these studies suggest that brain reward systems, including the VTA and its projections, play a critical and complex role in determining stress resilience or susceptibility through both functional activity and distinct structural alterations in interconnected brain regions. The findings also emphasize the importance of targeting maladaptive plasticity and disrupted interactions within these circuits for potential therapeutic intervention.

#### Genes and proteins

Several molecular and genetic mechanisms have been closely linked to resilience to chronic stress. One of these is related to the transcription factors DeltaFosB and DeltacJun, which interact with and regulate each other. For example, in mice exposed to a period of chronic SDS, both the core and shell subregions of the NAc of resilient animals showed a significantly greater increase in DeltaFosB than was seen in susceptible animals.^65^ A strong correlation also existed between levels of DeltaFosB and performance in social interaction paradigms, indicating that stress-induced levels of DeltaFosB may be a critical factor distinguishing resilient and susceptible phenotypes. Thus, DeltaFosB overexpression in the NAc protected against social avoidance, whereas its inhibition via DeltacJun overexpression increased susceptibility to social defeat.^65^ Of note, DeltaFosB has also been heavily implicated in addiction and drug misuse-related processes. Previous studies found that DeltaFosB accumulated in the NAc following repeated administration of multiple drugs of abuse, including alcohol.^66^ This accumulation can result in persistent changes in gene expression, because DeltaFosB is extremely stable and can remain in neurons for weeks after the final drug administration. Given the low baseline levels of DeltaFosB expression after prolonged social defeat, stress-susceptible animals may experience a more significant increase in DeltaFosB levels upon exposure to drugs of abuse, rendering them more vulnerable to addiction compared to resilient animals. Therefore, DeltaFosB may serve as a “molecular switch” for sustaining an addicted state.^67^

In addition to these transcriptional mechanisms, molecules related to synaptic plasticity have also been implicated in stress resilience and susceptibility. Glutamate receptor 2 (GluR2)—a subunit of the alpha-amino-3-hydroxy-5-methyl-4-isoxazolepropionic acid (AMPA) receptor—was upregulated in social stress-resilient mice, whereas susceptible mice exhibited a lower GluR2 ratio, contributing to maladaptive stress responses.^68^ Targeted modulation of AMPA receptor activity, either through receptor antagonists or genetic manipulation of GluR2, was able to reverse social avoidance and promote resilience, highlighting the critical role of glutamatergic signaling in stress-coping mechanisms, with potential therapeutic implications for stress-related disorders.

Another potential molecule that may contribute to variation in stress susceptibility and resilience is FK506 binding protein 5 (FKBP5), which can lead to dysregulation of the HPA axis. FKBP5 plays a significant role in the body’s stress response system, particularly in regulating how the body responds to cortisol in humans or corticosterone in rodents. Clinical studies indicated that FKBP5 dysregulation was prevalent in individuals with PTSD, and some gene variants increased susceptibility to PTSD after trauma exposure.^69^ Preclinical studies have also identified FKBP5 as a viable future target for the treatment of comorbid PTSD and AUD. In a rat model of this comorbidity that leveraged the “two-hit” stress model, FKBP5 inhibitors impacted both stress-related behaviors and alcohol consumption as determined by a two-bottle choice paradigm in adult male and female rats.^70^ Benztropine, an FDA-approved drug that disrupts the association of FKBP5 and glucocorticoid receptor-heat shock protein 90 complexes, significantly reduced alcohol preference but not alcohol intake in shock-stressed males and females. Benztropine additionally reduced startle responses to a 120-dB stimulus in shock-stressed males and females.^70^ Another FKBP5 inhibitor, SAFit2, reduced alcohol intake in shock-stressed males, but not females. SAFit2 also similarly reduced 120 dB startle responses and decreased fear-generalization in both male and female rats. Together, these results validate the use of FKBP5 as both a biomarker and potential target for comorbid PTSD and AUD, with some sex-specific effects.^70^

Given that a combination of stress and alcohol could cause specific genetic and behavioral changes, a preclinical study examined the effects of combined stress and alcohol exposure on the central amygdala, a brain region crucial for emotional regulation. This combined exposure led to an increase in transcripts of stress-related genes, such as corticotropin-releasing hormone (CRH) and FKBP5, but a decrease in mRNAs of brain-derived neurotropic factor (BDNF) and IL-18 in the central amygdala.^71^ However, rats exposed only to stress without alcohol exposure showed no significant differences in mRNA levels of CRH, FKBP5, or BDNF in the central amygdala, suggesting that a combination of stress and alcohol exposure is required for the alteration of transcriptions in this brain region. These findings are particularly relevant because IL-18 is a key player in neuroinflammatory states, with imbalances linked to conditions such as PTSD, anxiety, and AUD,^72^ whereas BDNF is vital for cognitive flexibility, dendrite development, and synaptic growth, which are often impaired in these conditions.^73^

In the hippocampus, increased stress susceptibility was related to BDNF levels as evidenced by male mice with BDNF depletion showing higher vulnerability to chronic mild stress compared to controls.^54^ Similarly, in rats, those susceptible to chronic mild stress or inescapable foot shocks exhibited significantly lower BDNF levels in the dorsal hippocampus compared to resilient rats.^74^ BDNF also interacted with other resilience factors, such as branched-chain amino acids, which enhance stress resilience through upregulation of BDNF expression, and IL-4. Thus, increased hippocampal BDNF mediated the resilience-promoting effects of IL-4, including dampening harmful inflammatory processes, fostering a neuroprotective environment for microglia, and promoting neurogenesis.^55^, ^56^ These findings underscore BDNF’s essential role in adaptive stress response of the brain area crucial for neuronal plasticity.

In addition to chronic SDS, a recent study using a SEFL paradigm that mimics the development of a persistent traumatic memory showed unique molecular changes in stress-susceptible males, distinct from their stress-resilient male counterparts.^25^ Epigenetic profiling revealed differential expressions between susceptible and resilient animals in PTSD-linked genes, including *Adcyap1*, *Bdnf*, *Drd2*, and novel targets, such as tachykinin receptor 1 (*tacr1*, a receptor that binds to the neuropeptide substance P), which may contribute to stress responses yet remain underexplored in PTSD.^25^

#### Neuroendocrine systems

The HPA axis is a complex neuroendocrine system essential for regulating the body’s response to stress. Its activation is a common feature across nearly all stress models, and dysregulation of the HPA axis is a central feature of stress-related psychopathology. For example, stress-susceptible mice displayed a maladaptive stress response characterized by an overactive HPA axis as evidenced by increased c-Fos expression in the paraventricular hypothalamus, a key brain region that initiates the stress cascade.^49^ This prolonged or exaggerated activation of stress pathways can directly disrupt the brain’s reward pathways and may make the rewarding effects of alcohol more pronounced; it can also create a need to self-medicate to alleviate discomfort, likely contributing to increased vulnerability to stress-induced alcohol-seeking behavior.^18^

Variability in glucocorticoid receptor activity and corticosteroid signaling may contribute to individual differences in stress responses related to resilience and susceptibility. Corticosterone increases in resilient animals following predator stress suggested resilience mechanisms potentially buffering corticosteroid dysregulation.^23^ This was exhibited by 124% increases in plasma corticosterone levels in the resilient subgroup compared with nonstress-exposed animals, whereas the increase was 88% in the susceptible subgroup.^23^ Sex differences showing higher corticosterone levels after ethanol consumption suggested greater HPA axis dysregulation in females.^34^ These variations suggested an amplified neuroendocrine response in stress-susceptible individuals, likely driven by heightened activity in corticolimbic brain regions, such as the mPFC and hippocampus. Specifically, stress-susceptible females showed an upregulation of CRH receptors (CRH-R1, CRH-R2), glucocorticoid receptors, and CRH-binding protein in the mPFC, contrasting with the downregulation of these receptors in resilient males.^23^, ^34^ These findings highlight the sex and phenotype-specific regulation of stress responses at the receptor level.

Alongside CRH, a study found that NPY also appears to vary between resilient and susceptible mice.^57^ Typically, the NPY Y1 receptor (Y1R) reduces anxiety-like behaviors, whereas the NPY Y2 receptor (Y2R) increases anxiety-like behavior. However, in resilient subgroups, overexpression of Y2R decreased fear response, whereas stress-susceptible mice, which have increased anxiety-like behaviors, had a higher expression of Y1R in the basolateral amygdala. In the stress-resilient mice, Y2R expression was increased in the central amygdala, and NPY levels in the bed nucleus of the stria terminalis were higher than in control animals not exposed to stress.^57^ These findings indicate that the differential regulation of stress-related neuropeptide pathways plays a role in stress susceptibility and resilience.

In the context of social stress, shared neurobiological mechanisms involving the upregulation of arginine vasopressin (AVP) and oxytocin may play a significant role in the development of comorbid anxiety and AUD. Rats that were exposed to SDS and experienced stress through observation (witness stress), rather than direct SDS showed increased expression of AVP and oxytocin in the amygdala.^29^ This upregulation was greater in rats that displayed both increased anxiety and increased alcohol consumption. In SDS, the expression level of these genes positively correlated with the comorbidity index, with higher gene expression correlating with more pronounced anxiety and alcohol consumption.^29^ This correlation suggests a potential link between gene expression in the amygdala and the manifestation of comorbid behaviors. Additionally, while both SDS- and witness stress-exposed rats exhibited escalated alcohol use and anxiety, the gene expression patterns were distinct between the two stress groups, implying that different molecular pathways may underlie these similar behavioral outcomes.^29^ However, the shared upregulation of AVP and oxytocin across both stress groups suggests these neuropeptides may represent a convergent mechanism in stress-induced comorbidities.

In summary, the dysregulation of the HPA axis is a key element in how individuals respond to stress. Altered activities in key brain areas, such as the paraventricular hypothalamus and mPFC, suggest maladaptive stress response is tied to changes in emotional regulation and behaviors. In addition, variations in corticosterone levels and sex differences between resilient and susceptible subgroups highlight the individual differences in resilience mechanisms. Understanding the distinct responses among resilient and stress-susceptible individuals could guide therapeutic approaches, while the variations in neuroendocrine responses and receptor expression might serve as potential biomarkers for identifying individuals at risk of developing stress-related disorders.

#### Sex differences

Many neuropsychiatric disorders exhibit disproportionate prevalence ratios between men and women. For instance, neurodevelopmental disorders are more prevalent in males, whereas mood disorders, including anxiety, depression, and stress-related disorders, are more common in females.^75^, ^76^ Because women are nearly twice as likely as men to develop PTSD following a traumatic event and frequently experience distinct symptoms,^2^ investigations into female-specific comorbid AUD and PTSD may reveal new pathways and neurobiological mechanisms relevant to treatments. As summarized above, recent preclinical studies have illustrated the significance of examining sex-specific stress responses in animal models to better understand the underlying biology of these differences in stress and neuropsychiatric outcomes.

Upon exposure to a predator odor, rats display either defensive digging behavior (active coping) or increased immobility (passive coping). Male rats tended to have uniformly low digging behaviors and high immobility, indicative of passive coping. Females, by contrast, could be categorized into two subgroups: those with passive coping showed similar behaviors to the male rats, whereas those with active coping exhibited high digging and low immobility behaviors.^13^ In subsequent alcohol self-administration, the active-coping subgroup of female rats displayed an increase in alcohol intake compared to controls 30 days after exposure to predator odor. This contrasted with male rats and the passive-coping group of female rats, which did not show a significant increase in alcohol intake. Therefore, understanding coping strategies in the context of stress may clarify mechanisms underlying stress-induced alcohol consumption in both sexes.

Sex differences in corticosterone and stress responses additionally vary by rodent strain. For example, hypothalamic feedback dysregulates glucocorticoid signaling in genetically selected Marchigian Sardinian alcohol-preferring (msP) rats that display increased alcohol preference and elevated anxiety-like behaviors when compared to other rat strains; this makes these animals ideal candidates for studies of comorbid PTSD and AUD.^77^ Male and female msP rats, relative to Wistar rat controls, buried fewer marbles and showed higher levels of immobility in a previously exposed environment (i.e., the lower-arousal condition).^77^ In a novel context (i.e., the higher-arousal condition) both male and female msP rats buried a similar number of marbles compared to Wistar rats, but only female msP rats displayed higher levels of immobility.^77^ Following the marble-burying task, male and female Wistar and msP rats were treated with the synthetic glucocorticoid dexamethasone, which inhibits corticosterone/adrenocorticotropic hormone (ACTH) release through engagement of hypothalamic feedback processes.^77^ After vehicle or dexamethasone administration, rats were subjected to restraint stress or no stress. In males, basal corticosterone was slightly decreased in vehicle-treated msP rats relative to vehicle-treated Wistar rats.^77^ Dexamethasone decreased corticosterone levels across both strains; however, the impact of dexamethasone on circulating corticosterone levels was lower in msP animals.^77^

A similar interaction between genotype and drug was also seen with respect to ACTH levels. Basal ACTH levels were significantly higher in vehicle-treated msP rats, administration of dexamethasone reduced these levels, and restraint stress moderately increased ACTH levels.^77^ These observations suggest that HPA axis feedback was differently regulated in msP rats and Wistar rats under basal conditions. In addition, sex differences also appeared within the msP strain, with persistently higher corticosterone levels in females across all conditions when compared to Wistar females.^77^ Female msP rats also displayed a small increase in corticosterone following restraint stress. In contrast, ACTH levels were only significantly affected by drug type, and there were no differences in corticosterone:ACTH ratios across strains.^77^ This further emphasizes strain-dependent differences in HPA axis activation, with an underlying sex-dependent component. Moreover, dysregulated glucocorticoid receptor signaling was shown in msP rats in the paraventricular nucleus and central amygdala, which varied across sexes.^77^ These findings suggest a general decrease in the hypothalamic processes that regulate neuroendocrine responses to stress. Alterations in the HPA stress response in msP rats may additionally provide insight into the comorbid presentation of AUD and PTSD. Acute alcohol exposure resulted in increased release of corticosterone and ACTH, whereas chronic alcohol exposure and dependence resulted in dampened neuroendocrine responses.^78^ Therefore, dysregulation of stress responses, specifically corticosterone and glucocorticoid receptor function, may underlie both PTSD and AUD phenotypes in a sex-dependent manner.

## Conclusions

**Figure 1:**
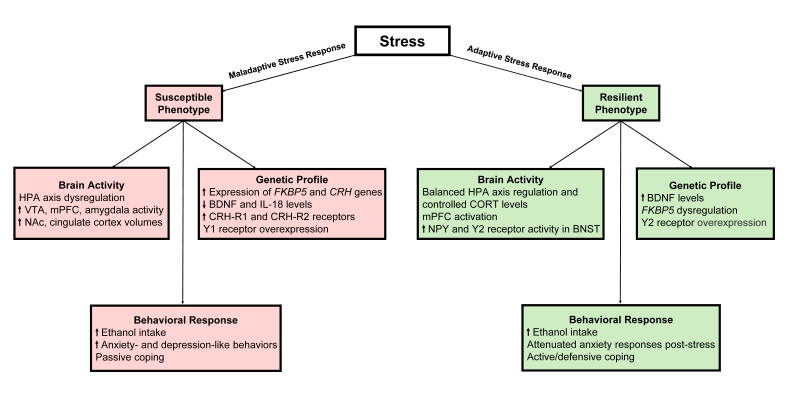
Genetic, neural, and behavioral changes related to stress susceptibility and resilience. *Note*: BDNF, brain-derived neurotropic factor; BNST, bed nucleus of the stria terminalis; CORT, corticosterone; CRH, corticotropin-releasing hormone; FKBP5, FK506-binding protein 5; HPA, hypothalamic-pituitary-adrenal gland; IL, interleukin; mPFC, medial prefrontal cortex; NAc, nucleus accumbens; NPY, neuropeptide Y; VTA, ventral tegmental area.

Clinical investigations of trauma and associated comorbid disorders, including AUD, are beginning to delineate a coherent array of elements and processes that contribute to stress resilience. Nevertheless, studies of the pretrauma period are limited and inadequately powered owing to minimal sample sizes. Consequently, preclinical investigations using animal models are poised to enhance understanding of the psychological, physiological, and neurobiological alterations throughout pre- and post-trauma phases. Given the heterogeneity of comorbid PTSD and AUD, employing various animal models of stress and alcohol drinking, alongside interdisciplinary approaches, is advantageous (see Figure 1). Examining sex differences in trauma responses and subsequent alcohol use also aids in identifying shared, translatable elements and processes that govern stress resilience and susceptibility. This increased knowledge could facilitate not only the development of tailored treatment plans that help individuals most affected by stress-related mental disorders but also aid in the identification of biomarkers that could aid in early diagnosis of PTSD. The identification of these markers is an ongoing process, yet it will be crucial for early diagnosis or risk assessment moving forward. In addition, these markers also may guide novel therapeutic efforts to promote mechanisms of natural resilience for at-risk individuals as preventative care.

## Figures and Tables

**Table 1:** Rodent Models of Stress

Stress Model	Description	Key Findings	Alcohol-Related Behaviors	Species/Strain	Citation
**Predator Stress**	Exposure to a predator-related odor, such as urine, or a synthetic odorant, such as trimethylthiazoline	Exposure reliably induced high arousal states, increased startle reactivity, and anxiety-like behaviors.	Animals with heightened anxiety or active avoidance behaviors show increased alcohol intake. Active-coping female rats increase alcohol intake following trimethylthiazoline exposure.	Male and female C57BL/6 mice; male and female Wistar rats	Wu (2013);^14^ Alavi (2022);^23^ Albrechet-Souza (2020)^24^
**Stress-Enhanced Fear Learning**	An initial stressor paired with subsequent auditory fear conditioning	Mice could be categorized into susceptible versus resilient based on freezing rates. Male susceptible versus resilient mice showed transcriptional divergence for *Adcyap1, Drd2, Bdnf*.		Male and female C57BL/6 mice	Daws (2017)^25^
		Susceptible rats exhibited increased fear generalization and anxiety-like behavior.	Susceptible alcohol-naïve male rats increased alcohol preference and intake after a stress experience.	Male and female Long-Evans rats	Gonzalez (2021)^26^
**“Two-Hit” Inhibitory Avoidance**	Exposure to a two-chamber apparatus, one paired with a single shock; secondary shock in a familiar or novel environment	Environment impacted anxiety measures and ethanol consumption in a sex-dependent manner. Vulnerable male alcohol-drinking rats showed high levels of fear-generalization.	Impacts of novel versus familiar environment for secondary stressor on subsequent ethanol consumption were sex dependent, with shocks in a novel environment increasing female intake and shocks in a familiar environment increasing male intake.	Male and female Wistar rats	Steinman (2021);^27^ Kirson (2021)^28^
**Social Defeat Stress**	Exposure to multiple bouts of fighting with a larger, more aggressive animal of the same species	Animals showed heightened anxiety, depression-like symptoms. Resilient animals showed attenuated stress under social stress conditions and reduced alcohol intake.	Susceptible animals exhibited escalated alcohol consumption.	Male Wistar rats	Barchiesi (2021);^29^ Golden (2011);^30^ Lu (2021)^31^
**Limited Bedding and Nesting**	Insufficient material for dam to build a nest, and pups are reared on wire flooring	Basal corticosterone levels were elevated in dam and pups by postnatal day 9. Adrenal glands showed hypertrophy in rat pups (Sprague Dawley and Long-Evans). Pups exhibited cognitive deficits, impaired social functions, increased anxiety and anhedonia, altered gut function, stress vulnerability.		Male and female Sprague Dawley rats; male and female C57BL/6 mice	Walker (2017)^32^
**Maternal Deprivation**	Separation of the mother and pups for single or repeated sessions during early life		Female mice with maternal deprivation showed increased alcohol-seeking behavior. Male and female mice showed higher breakpoints in a progressive ratio task.	Male and female C57BL/6 mice	Peñasco (2015)^33^
			Maternal deprivation-exposed rats escalate alcohol consumption following a period of alcohol deprivation and secondary stress.	Male and female Wistar rats	Walker (2017)^32^
**Two-Hit Model (Maternal Deprivation-Chronic Restraint)**	Exposure to maternal deprivation during development, and subsequent chronic restraint stress in adulthood	Maternal deprivation-exposed male rats were resilient to increases in corticosterone following restraint. Maternal deprivation-exposed female rats showed a significant increase in corticosterone levels following restraint.	Maternal deprivation-exposed rats lever-pressed significantly more to earn 10% ethanol in the operant condition. These animals showed an increase in consumption following chronic restraint but returned to their own relative baseline quickly.	Male and female Sprague Dawley rats	Bassey (2023)^34^

**Table 2:** Features of Susceptible and Resilient Phenotypes Regarding Alcohol Consumption After Stress Exposure

Key Concept	Susceptible Phenotype	Resilient Phenotype	Species/Strain	Citation
**Ethanol Consumption**	> 20% increase in ethanol intake following stress exposure Progressive escalation after multiple stress exposures Lower baseline ethanol intake	Stable or decreased ethanol intake following stress exposure Higher and more stable baseline ethanol intake	Male and female C57BL/6 mice	Alavi (2022)^23^
**Structural Differences**	Increased ventral tegmental area, amygdala, hippocampus, and hypothalamus volume Decreased cingulate cortex and nucleus accumbens volume	Balanced structural patterns Stronger hippocampus and hypothalamus connectivity	Male C57BL/6 mice	Anacker (2016)^53^
**Stress Response (Corticosterone)**	88% increase in plasma corticosterone levels following predator stress exposure relative to unexposed control animals	124% increase in plasma corticosterone levels following predator stress relative to unexposed control animals	Male and female C57B/6 mice	Alavi (2022)^23^
**Brain-Derived Neurotropic Factor (BDNF) and Stress**	Greater vulnerability to mild stress in BDNF-deficient male mice	Upregulation of BDNF and interleukin-4	Male and female Bdnf+/− and C56BL/ J mice Male Sprague Dawley rats Male CX_3_CR1^Cre/ERT2^ mice on a C57BL/6J background	Advani (2009);^54^ Nasrallah (2019);^55^ Zhang (2021)^56^
**HPA Axis and Brain Activation**	Increased c-Fos expression in paraventricular hypothalamus Higher c-Fos expression in medial prefrontal cortex following predator stress	Standard c-Fos expression, indicating normal stress response	Male and female C57BL6 mice	Alavi (2022)^23^
**Coping Strategies in Response to Stress**	Increased alcohol intake after predator stress in active coping subgroup (defensive digging behavior) in females	No significant increase in alcohol intake in passive coping subgroup (immobility) in females and passive coping males	Rats	Wu (2013);^14^ Kirson (2021)^28^
**Sex Differences in Stress and Alcohol Use**	Upregulation of corticotropin-releasing factor receptors (CRF-R1, CRF-R2), glucocorticoid receptors, and CRF-binding protein in the medial prefrontal cortex of stress-sensitive females	Down regulation of CRF-R1 and CRF-R2, glucocorticoid receptors in the mPFC in resilient males	Male and female C57BL/6 mice	Alavi (2022)^23^
**Neuropeptide Y (NPY)**	Higher expression of NPY receptor Y1R in the basolateral amygdala	Overexpression of NPY receptor Y2R, resulting in decreased fear response Increased Y2R expression in the central amygdala Higher NPY levels in the bed nucleus of the stria terminalis compared with control	Female Sprague Dawley rats	Denny (2021)^57^

*Note*: BDNF, brain-derived neurotropic factor; HPA, hypothalamic-pituitary-adrenal; mPFC, medial prefrontal cortex; NPY, neuropeptide Y.
